# Country-level perspectives and priorities to guide the development of Invasive non-Typhoidal *Salmonella* (iNTS) vaccines: Insights from seven countries

**DOI:** 10.1371/journal.pntd.0014012

**Published:** 2026-02-23

**Authors:** Dijana Spasenoska, Anna-Lea Kahn, Annelies Wilder-Smith, Adwoa D. Bentsi-Enchill, Yong-ha Hwang, Sajan Gunarathna, Jerome H. Kim, Jung-Seok Lee

**Affiliations:** 1 Department of Immunization, Vaccines and Biologicals, World Health Organization, Geneva, Switzerland; 2 Independent Consultant, Accra, Ghana; 3 International Vaccine Institute, Seoul, Republic of Korea; 4 College of Natural Sciences, Seoul National University, Seoul, Republic of Korea; Yale University School of Medicine, UNITED STATES OF AMERICA

## Abstract

Invasive non-typhoidal *Salmonella* (iNTS) disease typically presents as a non-specific febrile illness that can progress to bloodstream infections and carries a high case fatality rate. Early detection improves patient outcomes, however, in resource-constrained settings, limited diagnostic capacity leads to underdiagnosis, insufficient incidence data, and incomplete epidemiological and socio-economic research on iNTS. Although no licensed vaccines currently exist, several candidates are in pre-clinical and clinical development. In the early stages of vaccine development, it is essential to consider country-level perspectives and priorities to guide the development of novel vaccines. To support this, the World Health Organization’s Vaccine Innovation Framework was applied to conduct a consultation with 40 stakeholders representing the national immunization programmes in Burkina Faso, Ghana the Gambia, the Democratic Republic of the Congo (DRC), Kenya, Malawi and Mozambique. The consultation aimed to assess the stakeholder perceptions on the need for an iNTS vaccine and to evaluate its desirability, suitability, and evidence requirements from a low- and middle-income country (LMIC) perspective. Stakeholders argued that although an iNTS vaccines is desirable, the lack of nationally available epidemiological data is a barrier to accurately understanding the disease burden. A novel vaccine would be more desirable if it aligns with the preferred product characteristics specified, and helps mitigate, rather than exacerbate, current immunization programme challenges. They also expressed a preference for combination vaccines that align programmatically, including formulations combining iNTS with typhoid conjugate vaccine (TCV), to reduce the number of injections and simplify delivery logistics. To make evidence-based decisions on potential introduction, stakeholders emphasized the need for robust data on the value and impact of iNTS vaccines.

## Author summary

Invasive non-typhoidal *Salmonella* (iNTS) disease causes severe bloodstream infections, resulting in high mortality, especially among children, and in low income countries. Limited diagnostic capacity contributes to underdiagnosis and insufficient data on disease burden. Currently, there are several vaccine candidates in development. To inform early vaccine development, we applied the World Health Organization’s Vaccine Innovation Framework to consult 40 national stakeholders from Burkina Faso, the Gambia, the Democratic Republic of the Congo, Kenya, Malawi and Mozambique. Stakeholders argued that although iNTS vaccine is desirable, the lack of national epidemiological data is a key barrier to assessing true need. Stakeholders emphasized that given the increasing number of vaccines in the immunization schedule, they would prefer novel combination vaccines, including iNTS and typhoid conjugate vaccine (TCV). Engaging national stakeholders early in vaccine development helps ensure that future iNTS vaccines are aligned with the needs and priorities of the countries where the disease is most prevalent.

## Background

*Salmonella* are widely present in several animal species and their transmission through the food chain via contaminated food (e.g., meat, eggs) can cause human disease [1]. The species *S. enterica* is responsible for most of human disease, where serovars *S*. Typhi and *S.* Paratyphi A are associated with enteric fever (typhoid and paratyphoid fever respectively), while *S.* Typhimurium and *S*. Enteritidis are among the serovars associated with non-typhoidal *Salmonella* (NTS) [2]. NTS predominantly causes a self-limiting diarrheal illness in healthy individuals, while invasive non-typhoidal *Salmonella* (iNTS), most frequently caused by *S.* Typhimurium and *S*. Enteritidis, presents as bloodstream infection resulting in a case fatality of 20–25% [3].

The Global Burden of Disease data show that the global number of iNTS cases has increased by 45% since 1990 and reached 509,976 in 2021. Western sub-Saharan Africa has the highest number of cases, and children under the age of one have the highest incidence rate and number of deaths [4]. However, there are challenges in determining the true burden of disease due to lack of nationally reported data on incidence of disease or deaths caused by iNTS serovars [5]. Beyond human illness, iNTS infection imposes significant economic and societal cost to countries, but its impact is generally understudied. Direct medical costs per case reported in the literature are in the range of US$1,973–32,507 [6]. The societal impact of *Salmonella* infections includes adverse effects on productivity, child development and education, and household financial security [7].

Symptoms of iNTS disease are typically severe, non-specific febrile illness and respiratory symptoms clinically indistinguishable from other common childhood infections. Risk factors include HIV infection in adults, malaria, and malnutrition in children [3]. Although early detection and treatment are important for improving patient outcomes, a key challenge for diagnosis of iNTS disease is the lack of distinct clinical presentation and limited diagnostic capacity [8]. The surveillance standards by the World Health Organization (WHO) do not have a case definition for suspected iNTS disease due to the high degree of non-specificity in clinical presentation but state that it should be considered as a differential diagnosis in the presence of acute febrile illness in endemic settings, and confirmed through laboratory testing [9]. In the absence of a specific diagnosis, empiric treatment with antimicrobials is common. Yet, multi-drug resistance has been reported in nontyphoidal *Salmonella* isolates in sub-Saharan Africa [10]. The increase in antimicrobial resistance poses a global threat, which could result in significant morbidity and mortality, particularly in low-income countries, exacerbating the pressures on healthcare systems and the economic loss.

Currently, there is no licensed vaccine against non-typhoidal *Salmonella*. Vaccine candidates targeting iNTS disease are in pre-clinical and clinical development phases. They include bivalent vaccine candidates targeting *S.* Typhimurium and *S.* Enteritidis, and trivalent candidates which are co-formulated with a typhoid conjugate vaccine (TCV) to provide additional protection against *S.* Typhi [11,12]. Moreover, possible approaches to various combinations, such as iNTS and *Shigella* or iNTS and pneumococcal vaccines, as well as co-administration of iNTS and malaria vaccine have been considered [13]. In the recently published, preferred product characteristics (PPC) for iNTS vaccines, WHO highlights product development challenges including heterogeneous and incomplete epidemiological data, limited socio-economic research into the impact of iNTS disease, uncharacterised demand in low- and middle-income countries (LMICs) and uncertain commercial potential [14]. Consequently, there is a limited understanding of the potential public health value and market potential of iNTS vaccines.

To address some of these gaps, we used the WHO Vaccine Innovation Framework to conduct a consultation with national stakeholders from seven African countries. The Innovation Framework provides a structured approach to evidence-based discussions centred around credible and transparent consensus building [15]. The objectives of the consultation were to determine the need for iNTS vaccine as perceived by national stakeholders, to evaluate the desirability and suitability, and evidence needs for introduction of a novel iNTS vaccine from an LMIC perspective. The findings presented in this paper inform the iNTS Full Value of Vaccines Assessment [16] and offer insights into desirability of novel vaccines by national stakeholders.

## Methods

### Ethics statement

The generic protocol detailing the approach used for the workshops has been exempted by the WHO Research Ethics Review Committee (ERC) from requiring ethical review and clearance on the basis of the participating public officials being interviewed in their official, professional capacity on issues that are in the public domain. A statement of participation was presented at the beginning of the workshop and stakeholders were informed that by staying they consent to participating in the study.

We conducted the stakeholder consultation on iNTS vaccines in the African region, given that it bears the highest reported burden of disease [4]. Due to logistical constraints, the number of participating countries was limited to seven. These countries were selected based on their collective alignment with the following key criteria: data availability on iNTS disease incidence, sub-regional geographic diversity, progress towards transition from Gavi support to country-led vaccine financing, and TCV introduction status. In consultation with the WHO African Regional Office (WHO AFRO), as well as the respective country offices, Ghana was selected as a host-country for the workshop, and the additional six countries invited to send a delegation of relevant stakeholders were Burkina Faso, the Gambia, the Democratic Republic of the Congo (DRC), Kenya, Malawi and Mozambique. Together, these countries demonstrate diversity of combinations across the key criteria.

Each respective Ministry of Health was asked to nominate a delegation of three experts who could represent the following profiles: programmatic decision-makers from the Ministry of Health (e.g., immunization programme manager); policy decision-makers from the National Immunization Technical Advisory Group (NITAG); and service delivery professionals or logisticians involved in vaccination activities. In addition, a WHO Country Office technical officer was also part of the delegation for each country and WHO Regional focal points from each of the three sub-regional intercountry support teams were invited to take part. In total, 40 stakeholders attended the workshop, which was simultaneously conducted in English and French. Discussions were facilitated by a trained team from WHO and the International Vaccine Institute (IVI), who were provided facilitators’ instructions and Excel-based data collection tools for each session. Data were analysed descriptively. All participants were able to see the documented discussions during the workshop and propose changes if necessary.

We used the WHO Vaccine Innovation Framework to guide evidence-based discussions centered around credible and transparent consensus-building [15]. The discussions were systematically documented which allow reproducibility, consistency across countries and comparison of results. The Framework has previously been used for other innovations such as measles and rubella microarray patches, thermostable vaccines and oral cholera vaccine capsules [17–19].

**Fig 1:**
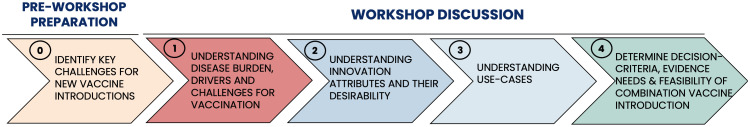
The steps of the WHO Vaccine Innovation Framework.

Relevant experts from WHO Headquarters and IVI jointly adapted the questions of the framework to iNTS vaccines, seeking inputs from topic experts for content validation. Fig 1 shows the steps of the framework.

Prior to the workshop, we conducted an initial desk review to identify the most common challenges for immunization programmes across the seven categories of the Essential Programme on Immunization (EPI): programme management and financing; human resources management; vaccine supply, quality and logistics; service delivery; immunization coverage and adverse events following immunization (AEFI) monitoring; disease surveillance and demand generation. The sources used included learnings from previous Innovation Framework Workshops for other innovations, common barriers reported for new vaccine introductions and barriers related to TCVs.

The workshop started with a series of technical presentations on the status of iNTS vaccine development and other relevant assessments to ensure all participants have equal understanding of the innovation discussed. Following the plenary, the workshop participants were divided into country-specific groups. During the first step of the Vaccine Innovation Framework, each group discussed the disease burden in their country and challenges related to the estimation of the burden, drivers and barriers to vaccination, and programmatic desirability of novel vaccines. They also assessed the relevance of the previously identified barriers within their national context by considering the impact of each barrier on coverage and equity, and the extent to which a given barrier could be related to iNTS. In step two, participants assessed the relevance of potential iNTS vaccine attributes, derived from the WHO preferred product characteristics for iNTS vaccines [14], to their national immunization programmes. Each group presented its findings in a plenary session through which consensus was then reached across the groups. Step three involved discussions on the desirability and feasibility of four potential use cases of the vaccine: bivalent iNTS vaccine as part of the EPI schedule; bivalent iNTS vaccine given at six months; trivalent iNTS vaccine (including TCV) for children; iNTS vaccine for adults with HIV. The questions addressed the desirability of the use case within the country, the main challenges to its adoption, potential mitigation strategies, and considerations for vaccine use in scenarios requiring multiple doses or boosters. In the final step, participants discussed their decision-making processes, relevant stakeholders to be involved in future vaccine introduction discussions and evidence needed for an informed decision. The discussions focused both on iNTS as a novel vaccine, as well as iNTS vaccine as a combination vaccine with other antigens or co-administration at the same touchpoint with other vaccines.

## Results

### Understanding the disease

Unexplained infant and child deaths exist in all countries, but blood culture tests are not routinely done due to lack of resources and lack of access to laboratories, preventing the actual burden of iNTS from being determined. The classification of those unexplained deaths varies between countries, and it is often based on clinical presentation rather than laboratory tests. Table 1 summarizes stakeholders’ views on how unexplained deaths are most commonly classified across countries.

**Table 1:** Most common explanations of unexplained infant deaths per country.

Country*	In your country, do you see deaths in infants that could not be explained?
**Burkina Faso**	Unexplained infant deaths exist. In the community they are attributed to malaria, or mystical power. Based on clinical manifestations, it is possible that some are undiagnosed iNTS, but this cannot be confirmed as diagnostics are not routinely performed.
**Ghana**	Unexplained infant deaths exist. Common clinical manifestations include hypoglycemia, dehydration, diarrhea, malnutrition. It is unknown if the deaths are undiagnosed iNTS in the absence of diagnostics and accurate case definition. General surveillance does not focus on iNTS, but in the country there is research-based iNTS surveillance.
**Democratic Republic of the Congo**	Unexplained infant deaths exist. In the community they are attributed to witchcraft/mysticism or malaria and typhoid without seeking confirmation. Based on clinical manifestations, some of those deaths might be caused by iNTS, but this cannot be confirmed as diagnostics are not available in most health facilities (with the exception of sentinel surveillance sites).
**Kenya**	Unexplained infant deaths exist. Those deaths are usually classified as acute febrile illness or death after short illness. Based on clinical manifestations, it is possible that some deaths are undiagnosed iNTS, but this cannot be confirmed as blood cultures are not collected, and there are no laboratory confirmations.
**Malawi**	Unexplained infant deaths exist. Commonly classified as fever, malaria, sepsis, diarrhoeal diseases. Based on clinical manifestations some of the deaths could be iNTS, but there is no routine iNTS surveillance or diagnostics. Research-based iNTS data available in the country (based at the Queen Elizabeth’s Central Hospital, Blantyre).
**The Gambia**	Unexplained infant deaths exist. Some classified as acute kidney injury. Based on clinical manifestations, some of those deaths could be iNTS, but there is no routine diagnosis. Survey data showed that iNTS deaths are often misclassified as pneumonia.

*Due to circumstances beyond their control, the delegation from Mozambique arrived late and did not participate in this part of the discussion.

Stakeholders explained that data on iNTS are not routinely collected in any of the countries. iNTS is not part of the surveillance system, and health workers are only partially aware of the disease. Currently, there is no clear standard case definition, and indicators are not incorporated into disease surveillance tools. Moreover, blood cultures are not routinely done, and even in cases of clinical presentation of the disease there is an absence of laboratory confirmation.

Data on iNTS are available from some countries through research activities, such as the studies conducted at the Queen Elizabeth Central Hospital in Blantyre, Malawi. However, not all stakeholders in each country are consistently aware of relevant research while it is being conducted, nor is this data systematically, effectively disseminated and brought to the attention of appropriate programme stakeholders. Workshop participants from The Gambia explained that a study published in 2021 based on population-based surveillance data between 2008–2016 found that the overall iNTS case fatality rate was 10%. Moreover, around half of the iNTS positive cases had been misdiagnosed as pneumonia cases [20]. Given the lack of data, it is difficult to determine with certainty the risk factors for iNTS. In The Gambia, acute or moderate malnutrition was present in iNTS culture positive cases. In Malawi, poor water and sanitation, HIV, malnutrition and low socio-economic status were identified as potential risk factors.

Stakeholders noted that lack of data has left country-level decision-makers only partially aware of iNTS disease, resulting in the absence of targeted measures to reduce its impact. This data gap might also hinder future decision-making regarding vaccine introduction, as this would center mainly around the burden of disease. While stakeholders believed that there was sufficient space in the immunization schedule for a new vaccine, determining the optimal timing for its introduction would be key.

Table 2 shows the list of immunization programme barriers which were identified prior to the workshop and their respective relevance to each country. It is evident that most of the barriers are relevant to all countries, albeit to a varying degree. The two barriers with the highest level of relevance are in the *Programme management and financing* category and relate to subnational budgeting and availability and disbursement of funds. The barriers relating to *Vaccine supply, quality and logistics* such as inadequate cold chain infrastructure and inappropriate monitoring of heat and freeze exposure were ranked as least relevant.

**Table 2:** List of barriers identified in desk review and their relevance to each country.

Country prioritization	Highly relevant		Slightly relevant		Not relevant	
**Potential barriers identified in a desk review**	Burkina Faso	Ghana	DRC	Kenya	Malawi	The Gambia
1.1 Inadequate subnational budgeting & disbursement of funds						
1.2 Delayed availability and disbursement of logistics at service delivery level						
2.1 Inadequate supply of health workers						
2.2 Suboptimal supervision of health workers						
2.3 Inadequate training of health workers						
3.1 Inadequate number of cold chain equipment						
3.2 Inappropriate monitoring of heat or freeze exposure during transport or storage						
3.3 Inadequate waste management						
4.1 Long distance, and lack of transport infrastructure limit service delivery						
4.2 Special and remote populations require more outreach efforts/resources						
4.3 Fragile or conflict settings disrupt immunization service delivery						
5.1 Challenges with denominators lead to inaccurate coverage estimates						
5.2 Lack of functional AEFI surveillance system						
6.1 Data in inadequate and not available in timely manner or not used for action						
7.1 Lack of confidence or fear of vaccines						
7.2 Inadequate social processes to encourage vaccination						

*Due to circumstances beyond their control, the delegation from Mozambique arrived late and did not participate in this part of the discussion.

** Abbreviations: AEFI (Adverse Events Following Immunization), DRC (the Democratic Republic of the Congo)

### Innovation attributes and their desirability

Thirteen attributes from the draft WHO PPC document for iNTS vaccines were presented to stakeholders. Those included indication, target population, dose regimen and schedule, safety, efficacy, duration of protection, immunogenicity, non-interference, administration, vaccine delivery strategy, product stability and storage, vaccine presentation and access and affordability. Participants determined that all of them are highly relevant in assessing the innovation’s desirability.

The first consideration by stakeholders was that a new vaccine would increase the number of injections given in the immunization programme and that it might introduce various new challenges such as an increase in the number of visits, logistical constraints, and reduction in demand due to fear of injections. Thus, it is important for the novel vaccine to result in the lowest number of injections and number of visits. For instance, single-dose vaccines are highly preferred, followed by a regimen that requires no more than two doses. The schedule should align with the existing immunization calendar, with a duration of protection of not less than two years.

The second consideration by stakeholders was the desired safety and efficacy of the novel vaccine. While acknowledging that data on iNTS are limited and the true burden could be underestimated, some stakeholders suggested that the incidence of iNTS in some countries may be relatively low compared to other leading causes of infant and child mortality. In the case of high-incidence disease, a low vaccine efficacy is not acceptable. Moreover, given the stakeholders’ interest in a potential trivalent iNTS + TCV vaccine, the efficacy of the new vaccine should not be lower than the licensed standalone TCVs. Similarly, the safety profile should be either better or similar to existing vaccines. If the safety profile is worse, the vaccine would not be desirable as it could increase vaccine hesitancy and reduce the demand for other vaccines as well.

The third consideration by stakeholders was their preference for a ready-to-use product that does not require reconstitution and is packaged in a multidose vial (ideally five doses) to simplify storage logistics. A thermostable product that could be used outside of the cold chain for 30 days was preferred; however, a product compatible with the standard cold chain would be acceptable. A product that would require procurement of different cold chain equipment is not desired.

Finally, the product is expected to be cost-effective and affordable. This is particularly a consideration for countries that are transitioning from Gavi support. Affordability of the vaccine would determine if long term use could be sustained, and whether high coverage and equity were to be achieved. The preferred vaccine delivery strategy would be through an initial one-time catch-up campaign followed by introduction in the routine vaccination schedule, similar to TCV.

### Use-cases

**Fig 2:**
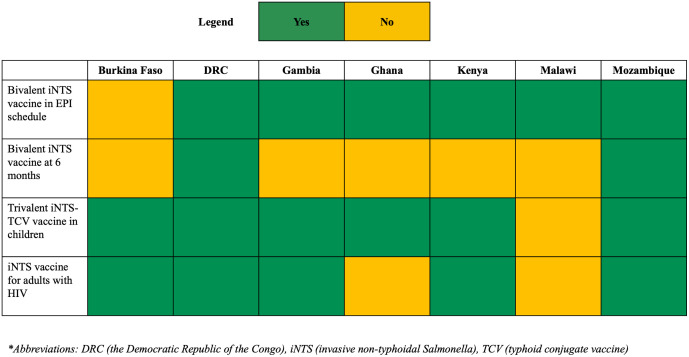
Would stakeholders select the use case for the introduction of iNTS in their country?. *Abbreviations: DRC (the Democratic Republic of the Congo), iNTS (invasive non-typhoidal Salmonella), TCV (typhoid conjugate vaccine).

All of the consulted stakeholders would use a bivalent iNTS vaccine in their national EPI schedule (Fig 2), with the exception of Burkina Faso whose delegation expressed a strong preference for a trivalent iNTS + TCV vaccine. The preferred timing for vaccine administration within the EPI schedule varied across countries. The Gambia favoured administration at four months of age, while Ghana preferred six weeks to align with existing vaccination touchpoints. Malawi proposed five months to enable co-administration with the recently introduced malaria vaccine. The main anticipated challenges relate to the increased number of injections and greater need for vaccine storage. These issues could be mitigated through the use of combination vaccine. Country stakeholders agreed that if a second dose is required, they would still consider the vaccine, but it is not preferred if it is beyond the first year of life.

Bivalent iNTS vaccine given at six months was found to be less desirable, and only DRC and Mozambique were open to adopting this use case. Mozambique specified that they would adopt this use case in the absence of trivalent vaccine. The main concern expressed by country stakeholders was that this use case introduces a new immunization touchpoint which would result in an increase in cost for both caregivers and the immunization programme. Moreover, they argued that given the age distribution of iNTS cases and their steep increase from six months, the vaccine should be given at an earlier age.

The use of trivalent vaccine (iNTS + TCV) in children was considered desirable by all countries except Malawi. Stakeholders highlighted that a combination vaccine could reduce the number of injections, improve acceptance, and optimize cold chain capacity. Nevertheless, stakeholders from Malawi expressed reservations, noting TCV has already been introduced at nine months. Introducing a trivalent vaccine would require changing the schedule, including the age at which children receive TCV.

Adults living with HIV are at a higher risk for iNTS infection. Stakeholders specified that should compelling data become available on iNTS-related morbidity and mortality among HIV-positive individuals, this population would be considered for vaccination as well. The target population could be reached through HIV clinics either upon diagnosis of the disease or later when treatment is distributed. However, numerous challenges were expected. First, HIV clinics are not attended by all HIV patients. Second, there might be stigma associated with HIV which would make it more difficult for patients to access the vaccine. Third, introducing the vaccine might increase burden on health workers in HIV clinics, and would require additional training. Finally, confusion might arise among HIV positive patients between treatment and vaccines. Thus, clear training for health workers and a carefully crafted advocacy strategy would be essential to ensure successful vaccine uptake.

### Understanding the decision pathway for iNTS vaccine introduction

The decision-making process for vaccine introductions primarily involves the Ministry of Health, NITAGs, partner organizations (e.g., WHO and UNICEF), and other relevant ministries. Multiple criteria are taken into account, including, by order of relevance, burden of disease, vaccine efficacy, logistics capacity, cost benefit/cost-effectiveness, safety, immunization schedule compatibility, immunogenicity, alignment with strategic plan, vaccine formulation, affordability and target population. Thus, to make informed decisions, evidence on iNTS vaccines is needed. Preferred studies include evidence on safety, disease burden, cost-effectiveness, efficacy, cold chain burden, programmatic feasibility (e.g., introduction pilots) and community acceptance studies (e.g., community interviews).

### Feasibility of delivering combination vaccines

Three potential vaccine combinations were discussed during the workshop: iNTS + TCV; iNTS + *Shigella* and iNTS + injectable rotavirus vaccine. Stakeholders identified the trivalent combination vaccine iNTS + TCV as more desirable than standalone iNTS vaccine. The most important criteria for decision-making distinguishing the two options included the burden of disease, safety, cost-effectiveness and programmatic suitability. The perceived benefits of such combinations include reductions in the vaccine delivery costs, reduction in the cold chain needs and number of injections and increases in the community acceptance. However, potential challenges were highlighted too. There were concerns regarding the target age cohorts for vaccine administration. Currently in Malawi, TCV is administered at nine months of age. Although licensed and recommended by the WHO Strategic Advisory Group of Experts on immunization that TCV can be given from the age of six months onward, changing the age at which the vaccine is given would require significant shift in the current operationalization. Furthermore, if the burden of one of the diseases is lower, especially if that is iNTS, it would be challenging to advocate to use the combination vaccine if that results in higher costs compared to the standalone TCV.

Stakeholders found the iNTS + *Shigella* vaccine combination to be somewhat desirable. Such a combination vaccine could simultaneously reduce the incidence of both diseases, offering a greater public health value. While the burden of iNTS disease is often underestimated, the more visible burden of *Shigella* could increase community acceptance of the vaccine. Moreover, this would be a new vaccine for both diseases, thereby eliminating any need for switches. However, to assess the need and desirability of such a vaccine, countries must strengthen diagnostic capacity. Accurately determining the burden of disease would support advocacy for the combination vaccine and would result in a higher political will to introduce it.

Stakeholders indicated that the pairing of iNTS with injectable rotavirus vaccine is not very desirable. The oral rotavirus vaccine is preferred by both health workers and caregivers, and a switch to an injectable vaccine would require it to be significantly better than the current vaccine. Moreover, based on the age distribution of the diseases, stakeholders argued that the two schedules are not compatible.

Other combination vaccines considered by stakeholders included iNTS and the pneumococcal vaccine (PCV) and penta/hexavalent vaccines. However, they argued that the age of administration in the schedule of these vaccines is very early, causing a lack of scheduling compatibility.

Stakeholders discussed the feasibility of co-administration of iNTS vaccine with other vaccines, including a malaria vaccine, meaning both vaccines administered at the same session but as two separate injections. The three most important criteria selected for making a decision around co-administration of vaccines included the burden of disease, programmatic suitability and cost effectiveness. Vaccines that appeal most for co-administration include malaria at six months and measles and yellow fever at nine months. In general, combination vaccines are more appealing than co-administration of vaccines. The benefits of co-administration include the possibility to leverage the existing schedule, to reduce delivery costs, and avoid repeating visits of vaccination sites. However, there are numerous challenges including the increase in the number of injections in a single visit which could increase vaccine hesitancy due to fear of multiple injections, as well as the burden on health workers. It was stressed that multiple injections in one visit is a significant barrier that could lead to low vaccine acceptability.

## Discussion

Stakeholders from Burkina Faso, Ghana, The Gambia, the DRC, Kenya, Malawi and Mozambique have indicated that there are clear gaps in routine data collection and surveillance for iNTS data at national level. This is primarily due to lack of a specific case definition in surveillance standards and lack of or inadequate diagnostic capacity in countries, in particular lack of blood culture facilities. Therefore, iNTS disease is typically under-reported and the burden of disease is underestimated. They concluded that a new iNTS vaccine is desirable, but its uptake would be dependent on the perceived need for it, and thus future decisions require accurate data on the burden of disease.

The lack of data on the prevalence and burden of iNTS hinders potential decision-making pathways. Stakeholders agreed that there is space in the immunization schedule for a new vaccine, yet they cautioned that in the context of increasing number of vaccine introductions the political will and/or resources available might be limited. Thus, beyond data on burden of diseases, the cost-effectiveness and the value of the vaccines to the society need to be demonstrated. While existing evidence suggests that there is a need for a vaccine against iNTS [21], conducting a full value of vaccine assessment would be appropriate to improve decision-making and communication among stakeholders involved in the decision-making pathways once the vaccine is available on the market [22].

Combination vaccines, including iNTS + TCV, were preferred over a standalone iNTS vaccine. This preference was mainly driven by the interest in reducing the number of touch points with beneficiaries, simplifying logistics and reducing the number of injections during one session. The benefits of combination vaccines recognized by national stakeholders are consistent with the potential value outlined for these vaccines [23]. However, it is important to highlight that in the countries represented during the workshop, both iNTS and typhoid disease are prevalent. In other settings such as countries in Asia, the iNTS prevalence is very low and thus in those settings a standalone vaccines might be more suitable [24].

Workshop discussions revealed that vaccine desirability is linked to how closely the novel vaccine would align with the characteristics outlined in the WHO PPC. Given noted challenges within immunization programmes, a novel vaccine product is expected to alleviate these issues rather than introduce new ones. For instance, the vaccine safety profile should be comparable to or better than similar existing vaccines, as reduced safety could negatively impact demand generation and acceptability, potentially undermining the broader EPI programme. Thermostability, especially the potential for use outside of the cold chain, could help overcome barriers associated with service delivery in remote settings. Furthermore, a preferred vaccine presentation would be a multi-dose vial, which minimizes storage space requirements and avoids placing further strain on the already burdened waste management systems.

Finally, it is important to acknowledge the limitations of the consultation findings. First, the discussions were based on a hypothetical iNTS vaccine, as defined in the preferred product characteristics. As such, there was an underlying assumption among stakeholders that a novel vaccine would meet the optimal characteristics, and the applicability of these findings to a novel vaccine that greatly diverges from the preferred product characteristics is limited. Second, stakeholders noted the limited data availability and knowledge around iNTS at the country level, meaning their views could evolve as more data become available, especially if the burden of disease proves higher than what they have assumed, thereby increasing the desirability for the vaccine or vice versa. Third, the results represent the views of the stakeholders present at the workshop, and do not imply any commitment from the countries to adopt the novel product in the future. Moreover, given the early stages of product development, the specific logistical and operational pathways for implementation were not explored. Instead, feasibility was assessed based on participating stakeholder experience. Lastly, due to logistical constraints, the number of stakeholders present at the workshop was limited, thus other perspectives from other sectors of the health system, geographical locations, or civil society might not have been captured. Nevertheless, the structured methodology used in this workshop allows for a systematic and robust documentation, making the process reproducible with different stakeholder groups, if needed in the future.

## Conclusion

Stakeholders from seven countries confirmed that an iNTS vaccine is considered desirable. However, the lack of nationally available data on the burden of iNTS disease, poses a challenge in assessing the public health value of such a vaccine. Stakeholders expressed a preference for a novel vaccine that would meet the preferred product characteristics and would not increase existing immunization programme barriers. The perspectives of national stakeholders should be at the forefront of vaccine development, ensuring that future iNTS vaccines are suitable and desirable for use in countries where the disease is prevalent.
